# The dilemma of xenotransplantation.

**DOI:** 10.3201/eid0202.960219

**Published:** 1996

**Authors:** C E Chastel


### The Dilemma of Xenotransplantation

**To the Editor:** I read with considerable interest Robert E. Michler's commentary on xenotransplantation (1).

From my point of view, that of a basic virologist, the dilemma is not to know in what "foreseeable future, clinical xenotransplantation may achieve its targeted goal of extended graft survival," but what deadly emerging infectious disease, most probably viral in nature, would arise in a recipient of a baboon or chimpanzee heart. While we face the terrific threat of AIDS, which clearly emerged from Africa and non-human primates 40 to 50 years ago, we are preparing a new infectious "Chernobyl."

Monkeys and apes harbor approximately 50 simian viruses; some of them pose a serious threat to humans, especially the heavily immunosuppressed. Recently, an outbreak of encephalitis related to a new type of reovirus (2) occurred among baboons from a colony used in human organ transplants. Moreover, once unknown or unrecognized simian viruses, like HIV, may be efficient invaders of the entire earth's population.

Xenotransplantation does not simply pose an ethical problem; it concerns the survival of the human species, an endangered species if transplant practitioners continue their course. Ronald Montalero, a virologist, was right when he said "unknown viruses were always a major concern in xenotransplants" (2). A moratorium on these procedures seems the best solution until all simian pathogens are identified and the risks they pose to humans are clearly established.

**Claude E. Chastel**
Virus Laboratory, Faculty of Medicine,
29285 Brest Cedex, France

### References

1. Michler RE. Xenotransplantation: risks, clinical potential, and future prospects. Emerging Infectious Diseases 1996; 2; 64-70.
2. Mystery virus fells donor baboons. Science 1994; 264; 1523.

### The Thucydides Syndrome: Ebola Déjà Vu? (or Ebola Reemergent?)

**To the Editor**: The plague of Athens (430-427/425 B.C.) persists as one of the great medical mysteries of antiquity (1-5). Sometimes termed "the Thucydides syndrome" for the evocative narrative provided by that contemporary observer (6, 7), the plague of Athens has been the subject of conjecture for centuries. In an unprecedented, devastating 3-year appearance, the disease marked the end of the Age of Pericles in Athens and, as much as the war with Sparta, it may have hastened the end of the Golden Age of Greece (3). Understood by Thucydides to have its origin "in Ethiopia beyond Egypt, it next descended into Egypt and Libya" and then "suddenly fell upon" Athens' walled port Piraeus and then the city itself; there it ravaged the densely packed wartime populace of citizens, allies, and refugees. Thucydides, himself a surviving victim, notes that the year had been "especially free of disease" and describes the following major findings: After its "abrupt onset, persons in good health were seized first with strong fevers, redness and burning of the eyes, and the inside of the mouth, both the throat and tongue, immediately was bloody-looking and expelled an unusually foul breath. Following these came sneezing, hoarseness . . . a powerful cough . . . and every kind of bilious vomiting . . . and in most cases an empty heaving ensued that produced a strong spasm that ended quickly or lasted quite a while." The flesh, although neither especially hot nor pale, was "reddish, livid, and budding out in small blisters and ulcers." Subject to unquenchable thirst, victims suffered such high temperatures as to reject even the lightest coverings. Most perished "on the ninth or seventh day . . . with some strength still left or many later died of weakness once the sickness passed down into the bowels, where the ulceration became violent and extreme diarrhea simultaneously laid hold (2.49)." Those who survived became immune, but those who vainly attended or even visited the sick fell victim (2.51).

By comparison, a modern case definition of Ebola virus infection notes sudden onset, fever, headache, and pharyngitis, followed by cough, vomiting, diarrhea, maculopapular rash, and hemorrhagic diathesis, with a case-fatality rate of 50% to 90%, death typically occurring in the second week of the disease. Disease among health-care providers and care givers has been a prominent feature (8, 9). In a review of the 1995 Ebola outbreak in Zaire, the Centers for Disease

